# The Effect of Menopausal Symptoms on Subjective Well-Being

**DOI:** 10.3390/healthcare14111436

**Published:** 2026-05-22

**Authors:** Derya Yuksel Koçak, Cem Koçak

**Affiliations:** 1 Nursing Department, Faculty of Health Sciences, Hitit University, 19200 Çorum, Turkey; 2 Economics Department, Faculty of Economics and Administrative Sciences Economics, Hitit University, 19040 Çorum, Turkey; cemkocak@hitit.edu.tr

**Keywords:** menopause, menopausal symptoms, well-being, correlation analysis, regression analysis, nursing, Turkey

## Abstract

**Background:** Menopausal symptoms may adversely affect women’s overall health and well-being. **Aim:** This study investigated the effects of menopausal symptoms on subjective well-being in women in the 40–65 age group. **Methods:** The study sample consisted of 510 women, with 318 postmenopausal and 192 perimenopausal participants. Data were gathered using a Sociodemographic Information Form, the Menopause Rating Scale (MRS), and the Subjective Well-being Scale (SWBS), all administered as self-report instruments. Menopausal status was determined using the Stages of Reproductive Aging Workshop +10 criteria. Descriptive statistics, Chi-square test, Pearson correlation, and regression analyses were used. **Results:** Three regression models were specified to investigate the relationship between menopausal symptoms and subjective well-being. Model 1 demonstrated that overall menopausal symptoms were significant negative predictors of subjective well-being (B = −0.749, SE = 0.156, β = −0.260, t = −4.788, *p* < 0.001, 95% CI [−1.06, −0.44], R^2^ = 0.068). Model 2 showed that both urogenital symptoms (B = −1.208, SE = 0.517, β = −0.139, t = −2.336, *p* = 0.020, 95% CI [−2.22, −0.20]) and somatic symptoms (B = −2.068, SE = 0.731, β = −0.168, t = −2.830, *p* = 0.005, 95% CI [−3.50, −0.64]) were significant negative predictors. Model 3 indicated that psychological symptoms significantly and negatively predicted subjective well-being (B = −1.114, SE = 0.262, β = −0.233, t = −4.253, *p* < 0.001, 95% CI [−1.63, −0.60], R^2^ = 0.054). **Conclusions:** The findings highlight the importance of comprehensive health strategies and demonstrate that psychological symptoms significantly impact overall well-being.

## 1. Introduction

Menopause is defined as “cessation of menstruation due to the decline in ovarian function” [1]. Menopause is defined by the onset of amenorrhea persisting for 12 consecutive months. The postmenopausal period refers to the 6 to 8 years following menopause and extends until approximately age 65 [2]. With the increase in life expectancy at birth worldwide, the population of women in the perimenopausal or postmenopausal stages is projected to rise [1].

The menopausal period is related to many somatic, psychological, and urogenital complaints that may adversely affect overall health [3]. The severity of menopausal symptoms is affected by sociodemographic, gynecological, and cultural characteristics [4].

These symptoms not only cause physical discomfort but also diminish the quality of life and well-being of menopausal women [5]. Many factors affect a woman’s quality of life during menopause [6]. Evidence indicates that the increasing severity of menopausal symptoms is associated with a decline in quality of life [7,8,9,10].

Well-being is an important component of quality of life [11]. Subjective well-being (SWB) refers to individuals’ evaluations and interpretations of their own lives. This concept typically encompasses life satisfaction, positive assessments, and negative emotional responses [12].

SWB is understood as a cognitive state or attitude concerning an individual’s overall life. In this context, well-being is defined as “a person positively evaluating, approving of, or having a favorable view of their own life [13].

SWB is influenced to a moderate extent by heritable factors and is also associated with variables such as income level, living conditions, parenthood, and marital harmony [13]. The findings suggest a significant relationship between subjective well-being and quality of life. Data from 2533 adults across 11 countries show that, although life quality and well-being have distinct theoretical foundations, they converge empirically [14].

The findings of this study may be interpreted through the Diener Subjective Well-Being Model, which conceptualizes well-being as comprising cognitive evaluations of life satisfaction and affective experiences. In this context, menopausal symptoms can influence both emotional states and overall life satisfaction, thereby affecting subjective well-being.

Studies investigating the interrelation in the menopausal period and mental well-being indicate that psychological well-being is linked to psychosomatic symptoms, a history of premenstrual symptoms, general health status, and various lifestyle factors, such as attitudes toward aging and menopause, smoking, exercise, marital status, and interpersonal stress [15]. A recent two-stage study found that women at all stages of menopause reported greater optimism about their future during the first stage than during the second stage. Furthermore, perimenopausal and postmenopausal women reported fewer positive perceptions of their social roles compared to premenopausal women [16]. However, the effect of menopause-related symptoms on subjective well-being is not yet fully understood. While many studies emphasize the negative aspects of menopause, emerging perspectives indicate that menopause may also yield positive outcomes [16]. Given that intense symptoms are known to negatively affect quality of life, this study investigates the extent to which menopausal symptoms may also positively influence well-being.

Although subjective well-being is not a clinical diagnostic construct, it provides important complementary information beyond symptom-based measures. It reflects perceived quality of life and psychological adaptation and may therefore serve as a valuable patient-reported outcome for evaluating the broader impact of menopausal symptoms.

## 2. Materials and Methods

### 2.1. The Study Design and Participants

The present study investigated the impact of menopausal symptoms on subjective well-being. Using a cross-sectional design, 510 participants aged 40 to 65 years were assessed in person during outpatient visits at a hospital in the Central Black Sea region of Turkey between February and August 2022.

#### Study Hypotheses

**H0.**  *There is no significant relationship between menopausal symptoms and subjective well-being.*

**H1.**  *Subjective well-being decreases as the severity of menopausal symptoms increases.*

In a previous study, the mean score on the scale assessing menopausal symptoms in women was reported as 14.65 ± 7.62 [17]. Here, *n* denotes the number of individuals to be sampled.

Table t yielded a theoretical value of 1.96 at the specified degrees of freedom and the observed error level. σ is the universe standard. Deviation = 7.62 d = ± deviation (0.05) must be calculated based on how frequently the event occurs.

Sample size calculations indicated that at least 223 individuals were required to accurately represent the population. A total of 510 women participated in the study, including 318 postmenopausal and 192 perimenopausal individuals. Menopausal status was assessed using the Stages of Reproductive Aging Workshop +10 criteria [18].

Although the STRAW +10 criteria were used as a conceptual framework, menopausal status was determined based on self-reported menstrual patterns due to the lack of hormonal measurements. Therefore, classification relied on participants’ reports of menstrual regularity.

The inclusion criteria for this study were as follows: (1) being in the menopausal period, (2) willingness to participate, (3) absence of physical or mental disabilities, and (4) no known oncological disease. Written informed consent was obtained after researchers explained the study’s objectives to eligible participants. Following consent, a qualified researcher conducted structured, in-person interviews to collect data. Each interview lasted approximately twenty minutes.

The sample was limited to women who volunteered for the study. Menopausal status was included as an independent variable, while subjective well-being level served as the outcome variable. This research investigated well-being levels between perimenopausal and postmenopausal women, using menopausal status as the independent variable.

Regression analyses were conducted only in postmenopausal women, as no statistically significant correlations were found between menopausal symptoms and well-being in perimenopausal women. Therefore, regression modeling was not considered appropriate for the perimenopausal group.

A series of multiple linear regression analyses was conducted to examine the relationship between menopausal symptoms and subjective well-being. Subjective well-being (SWBS) was the dependent variable, and menopausal symptoms were the independent variables. Model 1 included the total score of the Menopause Rating Scale (MRS) as a predictor. Model 2 simultaneously entered the somatic and urogenital symptom subscales. Model 3 analyzed psychological symptoms as an independent predictor. All regression analyses were performed using the Enter method. Prior to analysis, assumptions of regression, including multicollinearity, normality of residuals, and the absence of outliers, were examined.

Regression analyses were performed using unadjusted models that included only menopausal symptom variables. Potential confounders, including hormone therapy use, sexual activity, parity, and socioeconomic status, were excluded due to a lack of available data. The assumptions of regression analysis, such as multicollinearity, normality of residuals, and the absence of influential outliers, were evaluated and found to be satisfied.

### 2.2. Ethical Considerations

All study procedures involving human participants were conducted in accordance with the ethical principles outlined in the Declaration of Helsinki. Prior to the commencement of the study, ethical approval was obtained from the Yozgat Bozok University Social and Human Sciences Ethics Committee (Date: 19 January 2022/No. 29-22). Written informed consent was obtained from all participants before their participation in the study.

### 2.3. Measures

Data collection of instruments included in the Sociodemographic Information Form, the Menopause Rating Scale (MRS), and the Subjective Well-Being Scale (SWBS).

The Sociodemographic Information Form comprised 13 items assessing age, education level, marital status, women’s and spouse’s educational and employment status, menopausal stage, smoking and alcohol use, body mass index (BMI), and presence of chronic disease.

The Menopause Rating Scale (MRS), developed by Schneider et al., is designed to assess the severity of menopausal symptoms. Gürkan conducted the Turkish validity and reliability study of this 11-item scale in 2004 [19]. The scale consists of three sub-dimensions: somatic, psychological, and urogenital symptoms. Each item is scored from 0 (none) to 4 (very severe), with higher scores reflecting greater symptom severity. In the present study, Cronbach’s alpha coefficient was 0.85.

The Subjective Well-Being Scale (SWBS), developed by Dost, assesses subjective well-being by measuring the frequency and intensity of positive and negative emotions, as well as individuals’ cognitive evaluations of their lives [20]. The scale includes subjective evaluations of living environments and reporting of both positive and negative emotions. Responses are recorded using a five-point Likert scale, where 5 represents “Completely Suitable,” and 1 represents “Not Suitable at all.” The total value on the scale spans from 46 to 230. The higher scores indicate greater subjective well-being [20]. Cronbach’s alpha coefficient was found to be 0.93 in this study.

### 2.4. Statistical Analysis

Descriptive statistics, Pearson correlation, and regression analysis were performed using SPSS 22.0 software in a digital environment for data evaluation. The statistically significant value was 0.05.

## 3. Results

### 3.1. Participant Characteristics

Most participants were postmenopausal (62.4%, *n* = 318), while 37.6% (*n* = 192) were perimenopausal. The average age of postmenopausal women was 53.19 ± 5.41 years, while that of perimenopausal women was 45.42 ± 4.37 years. There were significant differences between perimenopausal and postmenopausal women in terms of age, education level, marital status, employment status, spouse’s education and employment status, BMI, and having chronic disease (*p* ≤ 0.05) (Table 1). Postmenopausal women had a higher average age compared to perimenopausal women. The proportions of women with higher education, single status, retirement, housewife status, overweight, and chronic illness were greater among postmenopausal women than among perimenopausal women. Additionally, postmenopausal women showed a higher prevalence of spouses with primary-level education and retirement status (Table 1).

### 3.2. Frequency and Severity of Menopausal Symptoms

The total Menopause Rating Scale (MRS) score for women in the perimenopausal period was 17.93 ± 9.23, while the score for women in the postmenopausal period was 18.24 ± 8.84. The total score on the SWBS for perimenopausal women was 169.96 ± 25.43, compared to 166.03 ± 26.58 for postmenopausal women (Table 2).

### 3.3. Correlation Between MRS and WBS

Among postmenopausal women, a statistically significant inverse relationship was observed between subjective well-being and menopausal symptom severity (*p* < 0.01). Specifically, subjective well-being scores decrease as total MRS, somatic MRS, urogenital MRS, and psychological MRS scores increase in postmenopausal women (Table 3). Conversely, there was no statistically significant association between MRS and SWBS scores among perimenopausal women (*p* > 0.05).

### 3.4. Regression Analysis Utilizing MRS and WBS

Three regression models were developed to examine the structural relationships between menopausal symptoms and subjective well-being. All structural equation models and their corresponding regression coefficients, as shown in Table 4, are statistically significant (*p* ≤ 0.05). The following results are based on the postmenopausal group.

Due to the cross-sectional design of the study, causal relationships between menopausal symptoms and subjective well-being cannot be inferred. The findings should therefore be interpreted as associative rather than causal.

Model 1 indicates that the total Menopause Rating Scale (MRS) score accounts for 6.8% of the variance in subjective well-being among women experiencing menopause.

The combined effects of somatic, urogenital, and psychological symptoms accounted for 6.8% of the variance in subjective well-being in Model 1. Model 1 outlines a general structure for the negative effect of menopausal symptoms on subjective well-being (Table 4).

Model 2 demonstrates that somatic and urogenital menopausal symptoms negatively influence the subjective well-being of menopausal women, accounting for 6.6% of the variance.

Most of this effect is representing 4.7% of the variance. Specifically, somatic symptoms negatively affect subjective well-being by 3.8%, whereas urogenital symptoms contribute to a 2.9% negative effect. Model 2 indicates that subjective well-being is primarily influenced by somatic symptoms, with urogenital symptoms exerting a secondary effect (Table 4).

Model 3 demonstrates that psychological menopausal symptoms are negatively associated with subjective well-being, explaining 5.4% of the variance. This model specifically emphasizes the negative impact of psychological symptoms on subjective well-being (Table 4). Multicollinearity diagnostics indicated no violations of the assumptions, with VIF values ranging from 1.42 to 1.91.

The regression model was statistically significant, F (3, 506) = 7.284, *p* < 0.001, explaining 4.1% of the variance in subjective well-being. Somatic symptoms were a significant predictor of subjective well-being (β = −0.145, *p* = 0.005). Cook’s distance values ranged from 0.000 to 0.047, suggesting the absence of influential outliers in the dataset. Standardized residuals ranged from −2.97 to 2.30, further supporting the lack of extreme outliers and indicating an acceptable residual distribution.

## 4. Discussion

### 4.1. Mean MRS and SWBS Scores

Psychological symptoms were the most commonly reported among all participants in this study. The severity of menopausal symptoms was assessed, and women in both groups experienced moderate symptoms (Table 2). In contrast, previous studies have identified somatic symptoms as the most frequently reported, with psychosomatic and urogenital symptoms reported less often [21,22]. Variations in symptom prevalence may be attributable to the cultural characteristics of the sample group.

Perimenopausal women exhibit less severe symptoms than postmenopausal women when evaluated with the Menopause Rating Scale (MRS). Additionally, perimenopausal women report higher levels of subjective well-being than their postmenopausal counterparts.

### 4.2. An Analysis of the Relationship Between Subjective Well-Being and Menopausal Symptoms

The absence of a significant association in the perimenopausal group, together with the significant negative associations identified in the postmenopausal group, indicates that postmenopausal symptoms have a substantial impact on subjective well-being. The effect of symptoms on quality of life is well established [23,24,25]; the aim of our study is to identify which symptoms affect well-being during the menopausal period. No significant correlations were identified between menopausal symptoms and well-being among perimenopausal women. For this reason, regression analyses were limited to postmenopausal women. This may suggest that the relationship between menopausal symptoms and well-being becomes more pronounced after menopause, or that other factors may play a more dominant role during the perimenopausal period.

Regression analysis was employed to examine the effects of somatic, urogenital, and psychological menopausal symptoms on subjective well-being. Model 1 demonstrates that all menopausal symptoms negatively influence overall well-being. Menopausal symptoms are a significant negative predictor of subjective well-being. Greater severity of these symptoms correlates with reduced subjective well-being. Endocrine changes, particularly decreased levels of progesterone and estrogen, are associated with signs such as mood changes, hot flashes, sleep problems, night sweats, stress, depression, irritability, sexual dysfunction, and genitourinary disorders. Menopausal symptoms can act as catalysts, triggering additional symptoms. Overall, these symptoms are reported to have a significant negative impact on women’s well-being [26].

Model 2 demonstrates that somatic complaints are most significant factor affecting subjective well-being in menopausal women. Vasomotor symptoms (VMS) are the primary complaint reported in approximately 80% of women in menopause [27]. Previous research demonstrates a significant relationship between somatic symptoms and the well-being of menopausal women [21,28]. In terms of subjective well-being and health, caregivers in the clinic should not overlook somatic complaints in women. The findings suggest that both somatic and urogenital symptoms influence well-being. Evidence indicates a significant association between the intensity of somatic symptoms and the existence of urogenital symptoms, suggesting that women with more intense somatic complaints are more likely to report urogenital problems [21,29,30]. Systemic hormone replacement therapy (HRT) and localized estrogen therapy are primarily recommended for the management of urogenital symptoms, particularly vulvo-vaginal atrophy. Adding pelvic floor exercises may further improve clinical outcomes. Recent research demonstrated that Kegel exercises combined with intravaginal localized estrogen therapy alleviated moderate and severe vulvo-vaginal atrophy symptoms [31].

Model 3 indicates that psychological symptoms significantly reduce well-being. Menopause is linked to an increased risk of depressive symptoms, memory impairment, stress, attention deficits, and traumatic stress in women [32]. Psychological distress in the menopausal period may have a more significant effect on subjective well-being than physiological factors, including the menopausal phase or frequency of hot flashes [33]. Additionally, psychological problems such as anxiety, depression, and stress further reduce the well-being of women during menopause [34]. Psychosocial factors, including stress and anxiety, contribute to diminished subjective well-being by intensifying menopausal symptoms. Therefore, a bidirectional relationship exists between subjective well-being and psychological symptoms [35]. Menopausal symptoms adversely impact women’s subjective well-being and overall quality of life. Evidence indicates that interventions such as awareness training can improve psychological well-being by reducing symptom severity [36].

Regression models explained a small proportion of the variance in well-being (R^2^ = 0.054–0.068), suggesting that menopausal symptoms have a limited impact on subjective well-being. The low R^2^ values indicate that other factors beyond menopausal symptoms may play a more substantial role. Therefore, these results should be interpreted with caution.

### 4.3. Implications for Clinical Care

Clinical assessments of menopausal women in healthcare settings should encompass screening for physical, psychological, and urogenital symptoms. The use of standardized, reliable assessment tools is advised.

Interventions, including awareness training to support well-being during menopause, can reduce symptom severity and enhance psychological health.

Primary-care nurses are encouraged to inquire about urogenital symptoms, particularly in women presenting with vasomotor symptoms.

Healthcare professionals may recommend Kegel exercises alongside HRT for women experiencing urogenital symptoms of menopause. Nurses can act as trainers and consultants by instructing patients in these exercises, ensuring correct technique, and providing ongoing support.

### 4.4. Limitations

First, the cross-sectional design of this study precludes causal inference and allows only tentative interpretations of associations between variables. Additionally, hormonal levels, lifestyle factors, and cultural influences that may affect the menopause process were not incorporated into the model. Second, all data were obtained from self-reported questionnaires, which may introduce subjectivity and recall bias. Exclusive reliance on self-report scales (Menopause Rating Scale [MRS] and Subjective Well-Being Scale (SWBS) without objective or clinical validation further limits the robustness of the findings.

A further limitation of this study is that the regression analyses relied on unadjusted models that included only menopausal symptom variables. Key potential confounders, such as hormone therapy use, sexual activity, parity, and socioeconomic status, were not controlled due to the unavailability of data, which may have influenced the observed associations.

An additional limitation of this study is that regression analyses were performed exclusively on postmenopausal women. Limiting analyses to a single subgroup potentially reduces the generalizability of the findings. Subsequent research should examine these relationships across various menopausal stages.

## 5. Conclusions

In postmenopausal women, greater symptom severity correlates with reduced self-assessed quality of life. Somatic and urogenital symptoms have a more pronounced impact on well-being than psychological symptoms. These results highlight the importance of comprehensive health strategies and demonstrate that psychological symptoms also substantially influence overall well-being. Addressing both physical and psychological aspects of menopause may improve women’s overall happiness and satisfaction.

## Figures and Tables

**Table 1:** Distribution of perimenopausal and postmenopausal women by some characteristics.

Characteristics	Postmenopausal Women		Perimenopausal Women		Test Statistics *
	*n*	%	*n*	%	
Age (40–65)					
Between 40–48	92	28.9	173	90.1	*p* < 0.001
Between 49–57	183	57.5	13	6.8	
Between 58–65	43	13.5	6	3.1	
Mean	(min: 42–max: 65) 53.19 ± 5.41		(min: 40–max: 63) 45.42 ± 4.37		
Education level					
Literate—primary-school graduate	218	68.6	62	32.3	*p* < 0.001
Secondary school—high-school graduate	56	17.6	52	27.1	
College—university graduate	44	13.8	78	40.6	
Marital status					
Married	242	76.1	172	89.6	*p* < 0.001
Single	76	23.9	20	10.4	
Employment status					
Housewife	240	75.5	136	70.8	*p* = 0.013
Retired	34	10.7	12	6.3	
Employee	44	13.8	44	22.9	
Perception of income level					
Good	94	29.6	48	25.0	*p* = 0.499
Middle	196	61.6	124	64.6	
Low	28	8.8	20	10.4	
Spouse’s education level (*n* = 414)					
Literate—primary school graduate	150	61.5	69	40.6	*p* < 0.001
Secondary school—high-school graduate	64	26.2	55	32.4	
College—university graduate	30	12.3	46	27	
Spouse’s employment status (*n* = 414)					
Not working	8	3.2	6	3.5	*p* = 0.020
Retired	118	48.4	57	33.5	
Working	118	48.4	107	63.0	
Individuals living together					
Alone	160	50.3	92	47.9	*p* = 0.939
With spouse	127	39.9	82	42.7	
Spouse and children	20	6.3	12	6.3	
Family elders and children	11	3.5	6	3.1	
Smoking					
Smokers	38	11.9	28	14.6	*p* = 0.391
Non-smokers	280	88.1	164	85.4	
Alcohol use					
User	18	5.7	18	9.4	*p* = 0.113
Non-user	300	94.3	174	90.6	
BMI					
Normal	63	21.9	82	42.7	*p* < 0.001
Overweight	225	78.1	110	57.3	
Have a chronic disease					
Yes	218	68.6	114	59.4	*p* = 0.035
No	100	31.4	78	40.6	

* The chi-square.

**Table 2:** The mean scores of perimenopausal and postmenopausal women obtained from the Subjective Well-Being Scale (WBS) and Menopause Rating Scale (MRS).

Sub-Dimensions	Minimum-Maximum Scores *	Postmenopausal Women * X ± SD	Perimenopausal Women * X ± SD
Somatic	0–8	3.25 ± 2.40	3.33 ± 2.16
Urogenital	0–12	4.55 ± 3.13	3.99 ± 3.05
Psychological	0–24	10.44 ± 5.20	10.61 ± 5.55
MRS total	0–44	18.24 ± 8.84	17.93 ± 9.23
WBS total	46–230	166.03 ± 26.58	169.96 ± 25.43

* Descriptive statistics.

**Table 3:** Relationship between the scores of perimenopausal and postmenopausal women on Menopause Rating Scale (MRS) and Subjective Well-Being Scale (SWBS).

Variables	Postmenopausal Women SWBS		Perimenopausal Women SWBS	
	r	*p*	r	*p*
MRS	−0.260	*p* < 0.001 **	−0.051	0.484
Somatic	−0.223	*p* < 0.001 **	−0.131	0.070
Urogenital	−0.206	*p* < 0.001 **	−0.048	0.506
Psychological	−0.233	*p* < 0.001 **	0.003	0.966

** *p* < 0.01, Pearson’s correlation coefficient was.

**Table 4:** The Results of Regression Analysis for Post-menopausal women.

Regression Model	Independent Variables	Direct Effect	Total Effect
Model 1	WBS = 179.456 − 0.749 × MRS (*p* < 0.001)	MRS: 0.068	0.068
		R^2^ = 0.068	
Model 2	WBS = 177.747 − 2.068 × Somatic − 1.208 × Urogenital (*p* < 0.001; *p* = 0.005; *p* = 0.020)	Somatic: 0.028	0.038
		Urogenital: 0.019	0.029
		R^2^ = 0.066	
Model 3	WBS = 177.846 − 1.14 × Psychological (*p* < 0.001; *p* < 0.001)	Psychological: 0.054	0.054
		R^2^ = 0.054	

## Data Availability

The data presented in this study are available on request from the corresponding author. The data are not publicly available due to legal restriction.
